# Terbinafine Induced Lupus Erythematosus With Progression to Lupus Nephritis

**DOI:** 10.7759/cureus.23887

**Published:** 2022-04-06

**Authors:** Naif Hindosh, Ragarupa Kotala, Letty Probasco, Swomya Bal

**Affiliations:** 1 Internal Medicine, St. Luke's University Health Network, Easton, USA; 2 Internal medicine, St Luke's University Health Network, Easton, USA; 3 Nephrology, St Luke's University Health Network, Easton, USA

**Keywords:** lupus, acute cutaneous lupus erythematosus, terbinafine-induced dil, lupus nepritis, drug-induced lupus erythematosus

## Abstract

Drug-induced lupus erythematosus (DILE) is a syndrome that manifests with symptoms similar, but with less severity, to that of systemic lupus erythematosus (SLE). Many medications are reported to be involved in DILE; however, terbinafine (Lamisil) is not a well-known causative agent of this syndrome. In this case report, we present a 22-year-old male patient with no prior medical history presented with worsening fever, rash, joint pain, and weight loss a couple of weeks after starting terbinafine. He underwent an extensive workup which revealed worsening kidney function, proteinuria, and microscopic hematuria, for which he underwent a renal biopsy which revealed class IV lupus nephritis. He was treated with prednisone taper and immunosuppressants with subsequent resolution of his symptoms. Close monitoring for the development of DILE symptoms is recommended after starting terbinafine, especially in patients with a known personal or family history of SLE.

## Introduction

Multiple medications cause an autoimmune response with subsequent production of autoantibodies, manifesting clinically with symptoms similar to systemic lupus erythematosus (SLE). This clinical syndrome is referred to as drug-induced lupus erythematosus (DILE) [1], and it accounts for less than 15% of all causes of the spectrum of lupus. Approximately 15,000-30,000 DILE cases are reported in the United States annually [2]. While the exact cause of DILE is not well defined, multiple mechanisms have been involved. It is noted that the induction of autoimmunity in DILE is mediated by slow acetylators and certain genetic factors, including HLA- DR2, HLA-B8, DR3, HLA-DR4, and deficiency of complement system protein C4 [3-5]. Procainamide and hydralazine account for most DILE cases [2]. Other commonly prescribed medications that can cause DILE include quinidine, isoniazid, phenytoin, and minocycline. The clinical presentation of DILE differs depending on the causative agent; however, the most common presenting symptoms include arthralgia, fever, weight loss, and cutaneous manifestations such as pruritic rash and photosensitivity. DILE is rarely associated with renal and central nervous system involvement [6].

Terbinafine is an antifungal medication widely used to treat many dermatological diseases such as tinea infections, onychomycosis, and sporotrichosis. There have been a few cases reports in the medical literature demonstrating that this medication has been linked to DILE development. However, this association is not well known. We present a case of terbinafine-induced Lupus Nephritis (LN) Class IV in a young patient with no documented history of SLE.

## Case presentation

A 22-year-old-male with no significant past medical history presented to his primary care physician with multiple complaints, including low-grade fever, worsening rash over his arms and back, fatigue, myalgias, and a 15lb weight loss within four weeks. He began developing these symptoms approximately two weeks after starting a 6-week course of terbinafine prescribed for patchy scalp hair loss. He also took ibuprofen 400mg once daily for myalgia; however, he discontinued it after approximately three days because it was ineffective in relieving his symptoms. He denies recent infections, hospitalizations, tick bites, or sick contacts. He reported that his sister has a history of DILE secondary to minocycline; however, no other family member reported a similar condition. Vital signs were noted for a temperature of 98.9 °F (37.2 °C), 70 beats per minute, respiratory rate of 16 per minute, and blood pressure of 122/78 mm Hg. Physical examination was significant for a pink reticular macular rash on the back and upper extremities.

In addition, anterior cervical, submandibular, supraclavicular, and axillary lymphadenopathy was noted. Laboratory testing, including complete blood count (CBC), comprehensive metabolic panel (CMP), and serologic workup, are shown in Table 1 and Table 2. Serum creatinine noted to be 1.38mg/dL from a baseline of 1mg/dL. Urinalysis (UA), as shown in Table 3, demonstrated microscopic hematuria and proteinuria 100(+2) with a urine protein/creatinine ratio of 2.14. Skin biopsy was notable for focal vacuolar interface changes, and superficial perivascular lymphocytic infiltrate with focal increased dermal mucin deposition and absence of dermal infiltration of eosinophils or atypical lymphocytes. These histologic findings are non-specific and suggestive of connective tissue disease.

**Table 1:** Lab values MCV: mean corpuscular volume; BUN: blood urea nitrogen; eGFR: estimated glomerular filtration rate; AST: Aspartate aminotransferase; ALT: alanine aminotransferase

Test	Value	Reference range
Hemoglobin	11.4 g/dL	13.8 – 17.3 g/dL
MCV	93 fL	82-98 fL
White blood cells	4.82 Thousand/uL	4.00 – 10.40 Thousand/uL
Eosinophils Absolute	0.003 Thousand/µL	0- 0.61 Thousand/µL
Eosinophils Relative	1%	0 -6%
Platelets	187 Thousands/uL	141-377 Thousands/uL
Creatinine	1.38 mg/dL	0.60-1.30 mg/dL
BUN	30 mg / dL	5-25 mg/dL
eGFR	72 ml/min/1.73sq m	
AST	68 U/L	5-45 U/L
ALT	148 U/L	12-78 U/L
Alkaline phosphatase	65 U/L	46-116 U/L
Total bilirubin	0.68 mg/dL	0.20-1.00 mg/dL
Albumin	3.2 g/dL	3.5-5g/dL
Creatine kinase	44 U/L	24 - 195 U/L

**Table 2:** Serologic evaluation ANA: Antinuclear Antibody; CRP:C-reactive protein; ANCA: Anti-neutrophil cytoplasm antibodies

Test	Value	Reference range
Antinuclear Antibody (ANA)	Positive (1:640) with a homogenous pattern	Negative
Anti-DNase B antibody	199 U/mL	0-120 U/mL
Anti-DNA antibody, double-stranded	>300 IU/mL	0-9 IU/mL
Histone antibody	9.3	0.0 – 0.9
C3 Complement	21.1 mg/dL	90-180 mg/dL
C4 Complement	5.0 mg/dL	10.0-40.0 mg/dL
Total complement	<10 U/mL	>41 U/mL
C-reactive protein (CRP)	<3.0 mg/L	<3 mg/L
Sedimentation rate	31 mm/hour	0-14 mm/hour
Sjogren’s Antibodies SS-A (RO) Ab SS-B (LA) Ab	>8.0 Al 0.3 Al	0.0-0.9 Al 0.0-0.9 Al
Ant streptolysin O titer (ASO)	400 IU/ml	None
Rheumatoid factor	Negative	Negative
Anti-neutrophil cytoplasm antibodies (ANCA)	Negative	Negative

**Table 3:** Urine studies

Test	Value	Reference range
Protein	100mg/dl (+2)	Negative
Protein/creatinine ratio	2.14	0.00-0.10
RBC	4-10	0-4/hpf
Blood	Large	Negative
Specific gravity	1.005	1.003-1.030

**Figure 1:**
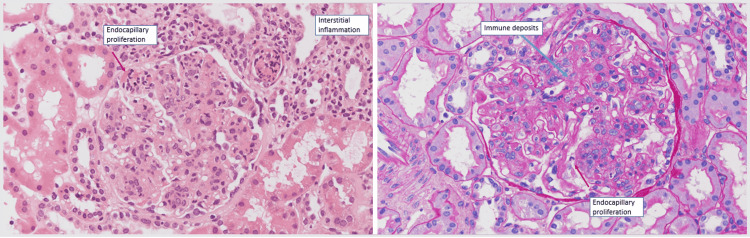
Microscopic findings of the renal biopsy: Glomeruli are enlarged and hypercellular with moderate diffuse mesangial hypercellularity. Glomeruli also display segmental to global endocapillary proliferation by infiltrating mononuclear leukocytes and neutrophils. Also noted segmental membranoproliferative features with large sub-endothelial immune deposits with mild interstitial inflammation by lymphocytes, monocytes, neutrophils, and rare eosinophils involving 10% of the cortex.

**Figure 2:**
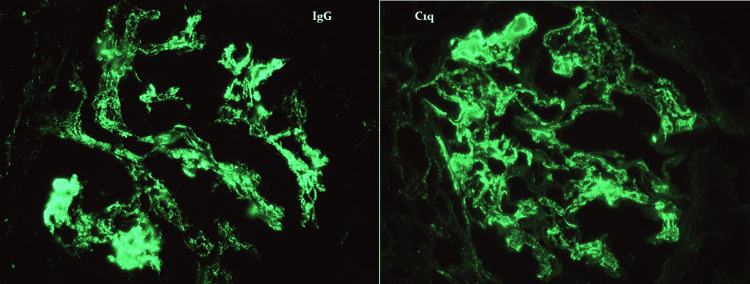
Immunofluorescence images

**Figure 3:**
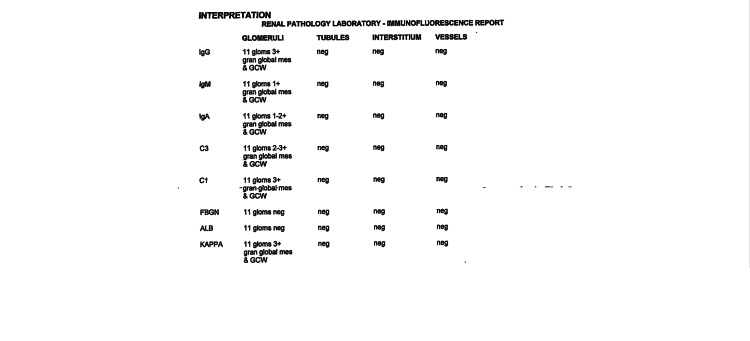
immunofluorescence report reveals a "full house" of immune staining and a granular global mesangial and glomerular capillary wall distribution.

Further testing included a negative mono test and positive Epstein Bar IgG; however, IgM was negative, consistent with prior infection. Lyme titer was negative, and serum protein electrophoresis showed no monoclonal bands. He was also tested for HIV and viral hepatitis A, B, and C, all negative. CT scan of the chest, abdomen, and pelvis was notable for a few mildly non-specific enlarged left axillary lymph nodes measuring up to 1.4 cm, otherwise was unremarkable. The patient was started initially on low-dose oral prednisone due to suspicion of autoimmune process, but with no clinical improvement. Due to persistent symptoms and worsening anemia and kidney functions, he was evaluated by nephrology. The patient underwent a renal biopsy which showed diffuse endocapillary and focal membranoproliferative glomerulonephritis, consistent with class IV lupus nephritis, as shown in Figure 1. Immunofluorescence revealed a "full house" of immune staining and a granular global mesangial and glomerular capillary distribution, supporting a diagnosis of immune complex-mediated glomerulonephritis as shown in Figures 2, 3.

After confirming the diagnosis of LN, the patient was started on Mycophenolate (Cellcept) 1.5gm twice daily, hydroxychloroquine (Plaquenil) 200mg twice daily, and prednisone 60 mg daily with tapering of 10 mg every two weeks. He was continued on Mycophenolate 1 gram twice daily as maintenance therapy for lupus nephritis with subsequent clinical improvement. Repeat laboratory testing was obtained frequently and was notable for significant improvement after three months of treatment, as shown in table 4.

**Table 4:** Lab values within 3 months of treatment eGFR: estimated glomerular filtration rate; AST: Aspartate aminotransferase; ALT: alanine aminotransferase

Test	Value	Reference range
Hemoglobin	12.9 g/dL	13.8 – 17.3 g/dL
Creatinine	1.17 mg/dL	0.60-1.30 mg/dL
eGFR	88 ml/min/1.73sq m	
AST	21 U/L	5-45 U/L
ALT	24 U/L	12-78 U/L
Albumin	4.2 g/dL	3.5-5g/dL
Anti-DNA antibody, double-stranded	21 IU/mL	0-9 IU/mL
C3 complement	71 mg/dL	90-180 mg/dL
C4 complement	16 mg/dL	10.0-40.0 mg/dL
Urine studies		
Protein	Trace	
Blood	4-10/hpf	0-4/hpf
Protein/Creatinine ratio	0.64	0.00-0.10

## Discussion

DILE is defined as a reaction induced by exposure to certain medications leading to lupus-like symptoms, which usually resolve following cessation of the offending agent. It often has a milder clinical presentation and similar serological findings to that of idiopathic systemic lupus erythematosus without its associated significant life-threatening complications. DILE is further classified into three major categories, which include SLE, subacute cutaneous lupus erythematosus (SCLE), and Chronic cutaneous lupus erythematosus (CCLE) [2].

To the best of our knowledge, only a few cases of Terbinafine-induced Lupus have been reported in the literature. The majority of cases already have a well-recognized history of SLE, making it difficult to distinguish if these cases were due to an SLE flare-up or a true DILE [6]. The average age of these patients was approximately 48 years, with 25-year-old being the youngest. In the prior case reports, it has been reported that the cutaneous manifestations appeared approximately 1-8 weeks after starting terbinafine [7]. In our patient, the cutaneous features started two weeks after starting the medication, consistent with what has been previously reported. Our patient, however, has no well-documented history of SLE before this episode. In terms of immunologic findings, Ro/SS-A autoantibodies were present in the majority of the previous case reports, while anti-histone antibodies were positive in one-third of them [6-7]. Both antibodies were positive in our patient. No previous case reports have demonstrated biopsy-proven lupus nephritis related to terbinafine exposure.

While renal involvement is a well-known feature of SLE, it remains a rare manifestation of DILE [8]. Kidney damage is usually seen initially or after a few months of SLE diagnosis, with most patients developing Lupus Nephritis (LN) within approximately six months to 5 years of diagnosis [9-10]. The pathogenesis of LN is multifactorial, with genetic and environmental components being described. Our patient's initial presentation showed renal involvement despite the lack of SLE diagnosis, raising the question if he already had undiagnosed SLE due to the absence of symptoms that were unmasked by the introduction of terbinafine or if the medication itself led to the induction of SLE with the rapid development of LN.

Different approaches have been used to treat DILE, including stopping the offending agent, prescribing topical steroids, oral prednisone tapering, dapsone, or other immunosuppressants like Mycophenolate and hydroxychloroquine [6-7]. Immunosuppressive medications are used to treat LN [11-12]. Our patient initially did not improve on low-dose prednisone therapy; however, significant improvement in his symptoms was noted one week after he was initiated on Mycophenolate (Cellcept) 1.5 grams twice daily hydroxychloroquine (Plaquenil) 200mg twice daily and high dose prednisone tapering. He was continued on Mycophenolate 1 gram twice daily as maintenance therapy for lupus nephritis. Most recent studies recommend that stopping the offending agent with concomitant use of steroids is usually enough to control symptoms of DILE [7]. Our patient required systemic immunosuppressants to control his symptoms. Our patient's treatment for lupus nephritis did not differ from what is reported in the literature.

Of note, there was a concern that the patient's clinical course could be related to drug reaction with eosinophilia and systemic symptoms (DRESS syndrome); however, in the absence of peripheral eosinophilia, high-grade fever, and the characteristic rash distribution in addition to non-specific skin biopsy results, this association felt to be less likely based on RegiSCAR study [13].

## Conclusions

Physicians and patients should be aware that terbinafine is a causative agent of DILE that could cause serious side effects, including progression to lupus nephritis. We recommend avoiding terbinafine and using Azole drugs as an alternative in patients with a known history of SLE; however, if terbinafine was used, then close monitoring of symptoms after initiating this medication is recommended. Immediate termination of the drug and symptoms-specific treatment is recommended when there is suspicion for DILE, assessing the need for further management with other systemic immunosuppressants depending on the clinical presentation. Renal biopsy with nephrology consultation is indicated when signs of kidney damage are noted clinically and on laboratory testing after initiating the medication to assess the possible development of lupus nephritis.
